# Correlation of sleep quality with fatigue and disease activity among patients with primary Sjögren’s syndrome: a cross-sectional study

**DOI:** 10.1590/1516-3180.2019.0251.R1.1912019

**Published:** 2020-03-09

**Authors:** Luciana Paula Dardin, Ana Beatriz Andreo Garcia, Fernanda Martins Gazoni, Fania Cristina dos Santos, Marco Tulio de Mello, Virginia Fernandes Moça Trevisani

**Affiliations:** I PT. Physiotherapist and Doctoral Student, Discipline of Emergency Medicine and Evidence-Based Medicine, Universidade Federal de São Paulo (UNIFESP), São Paulo (SP), Brazil.; II MD. Physician, Department of Ophthalmology, Universidade Federal de São Paulo (UNIFESP), São Paulo (SP), Brazil.; III MD. Physician, Discipline of Geriatrics and Gerontology, Universidade Federal de São Paulo (UNIFESP), São Paulo (SP), Brazil; and Doctoral Student, Discipline of Emergency Medicine and Evidence-Based Medicine, Universidade Federal de São Paulo (UNIFESP), São Paulo (SP), Brazil.; IV MD, PhD. Professor, Discipline of Geriatrics and Gerontology, Universidade Federal de São Paulo (UNIFESP), São Paulo (SP), Brazil.; V PhD. Professor, Physical Education School, Universidade Federal de Minas Gerais (UFMG), Belo Horizonte (MG), Brazil.; VI MD, PhD. Professor, Discipline of Emergency Medicine and Evidence-Based Medicine, Universidade Federal de São Paulo (UNIFESP), São Paulo (SP), Brazil; and Professor, Discipline of Rheumatology, Universidade Santo Amaro (UNISA), São Paulo (SP), Brazil.

**Keywords:** Sleep wake disorders, Fatigue, Sjogren’s syndrome., Sleep disturbance, Insomnia, Tiredness, Indisposition, Dry syndrome, Dryness.

## Abstract

**BACKGROUND:**

Fatigue is a frequent symptom in patients with primary Sjögren’s syndrome (pSS) and can be a cause of or be associated with sleep disorders.

**OBJECTIVE:**

To assess the sleep quality of pSS patients and its relationship with fatigue and disease activity.

**DESIGN AND SETTING:**

Analytical observational study conducted at an exercise psychobiology laboratory.

**METHODS:**

Sleep quality was evaluated using the Pittsburg sleep quality index (PSQI) and actigraphy. Fatigue was evaluated through the Profile of Fatigue and Discomfort - Sicca Symptoms Inventory (PROFAD-SSI-SF) and a visual analogue scale for fatigue (VAS-fatigue). Disease activity was evaluated using a visual analogue scale for pain (VAS-pain), EULAR Sjögren’s Syndrome Patient Reported Index (ESSPRI) and Disease Activity Index (ESSDAI). We summarized the data through descriptive statistics.

**RESULTS:**

A total of 50 female patients with pSS, of average age 56.4 years, were included in the study; 80% presented low disease activity. The total PSQI score showed that 74% had poor sleep. The actigraphy showed mean sleep latency of 26.2 minutes and mean nightly awakening of 48.2 minutes (duration of wakings after sleep onset, WASO). There were correlations between PSQI and VAS-pain, VAS-fatigue, PROFAD-SSI and ESSPRI. Actigraphy showed a correlation between the duration of WASO and ESSDAI.

**CONCLUSION:**

The present study provides important information regarding correlations between sleep disorders and disease activity. There is a need for proper control over disease activity and for development of strategies to help patients to sleep better in order to diminish their fatigue.

## INTRODUCTION

Sjögren’s syndrome (SS) is a systemic autoimmune disease that affects the exocrine glands and, less frequently, internal organs. It is characterized by intense lymphoplasmacytic infiltration, mainly in the epithelium of the tissues affected, and this leads to destruction and loss of their secreting function, and consequent xerostomia and keratoconjunctivitis.^1^^,^^2^

This syndrome can be seen alone, in which case it is known as primary Sjögren’s syndrome (pSS), or in association with other autoimmune disease such as rheumatoid arthritis, systemic lupus erythematosus or scleroderma, in which case it is classified as secondary Sjögren’s syndrome (sSS).^1^^,^^2^^,^^3^ These two variants of the syndrome are different with regard to their clinical, serological and immunogenetic aspects.^3^

Fatigue is a frequent symptom of pSS. It is considered to be a debilitating condition and is the most important cause of dysfunction in these patients.^4^^,^^5^^,^^6^ It has been described as a lack of physical or mental energy, i.e. a state of exhaustion, which interferes with the person’s ability to maintain his/her physical and cognitive activities. It can be persistent and severe.^4^^,^^5^^,^^6^ Several mechanisms have been proposed to explain occurrences of fatigue among pSS patients, but its underlying physiological basis remains insufficiently defined. It is thus a complex, multi-faceted and poorly understood phenomenon.^4^^,^^5^^,^^6^

In population-based studies, approximately 20% of healthy adults report experiencing fatigue and, among patients with autoimmune disorders, this percentage rises to 60%-70%.^1^ In pSS, fatigue is the most frequent non-exocrine symptom, and the prevalence of disabling fatigue among patients with pSS has been reported to be approximately 70%. It has been suggested that fatigue in pSS is mediated by the systemic inflammatory response that characterizes this syndrome. It has also been suggested that fatigue may be related to low blood pressure and abnormalities of the autonomic nervous system, sleep disorders, depression, sedentarism, comorbidities, disease activity, anemia, and decreased physical capacity.^7^^,^^8^^,^^9^^,^^10^^,^^11^^,^^12^

Wan-Fai and Simon^13^ suggested that fatigue in pSS may be associated with factors such as inflammation, sleep disorders, depression or dysfunction of the neuroendocrine and/or autonomic nervous system. Although the presence of sleep disorders in pSS patients has been previously confirmed in other studies, the relationship with fatigue and disease activity has been insufficiently studied.^7^^,^^14^

Insomnia can occur in approximately 33% to 50% of the adult population. Patients with chronic insomnia frequently report more feelings of fatigue (low energy, physical tiredness and weariness) than symptoms of sleepiness (i.e. a real tendency to fall asleep).^15^ Assessment of sleepiness among patients with sleep disorders should include use of questionnaires and clinical evaluations and a two-week sleep log to identify sleep patterns, such as through actigraphy.

The Pittsburgh sleep quality index (PSQI) is a self-report questionnaire that measures sleep quality on a Likert scale (0-3), in seven domains (subjective sleep quality, sleep latency, sleep duration, habitual sleep efficiency, sleep disturbances, use of sleep medication and daytime dysfunction over the last month). The sum of the scores for these domains yields one overall score, which ranges from 0 to 21, such that the highest score indicates worst sleep quality. Overall scores of 5 or greater indicate “poor” sleepers.^15^

Actigraphy is a valuable method for determining sleep patterns in normal, healthy adult populations and among patients who are suspected of having certain sleep disorders. Actigraphy enables recording of motor activity through limb movements. In comparison with polysomnography, it provides a reliability coefficient of 0.8-0.9 and is a less expensive method, although it cannot replace polysomnography. Several authors, including the American Academy of Sleep Medicine’s standards of practice committee, view actigraphy as a reliable method for assessing awakening patterns in adults.^16^^,^^17^ Actigraphy can be used easily, with the possibility of recording over many days. The primary baseline measurements obtained from a sleep log include, among others: bedtime, sleep latency (time taken to fall asleep), number of awakenings, duration of wakings after sleep onset (WASO: the sum of lengths of time spent awake between sleep onset and the final awakening), length of time spent in bed and total duration of sleep.^15^^,^^18^

## OBJECTIVE

The aim of the present study was to assess the sleep quality of patients with primary Sjögren’s syndrome (pSS) and its relationship with fatigue, quality of life and disease activity.

## METHODS

This was an observational, cross-sectional study in which participants in a clinical trial (NCT03130062) on pSS patients were evaluated. The clinical trial was conducted over a three-year period to evaluate the clinical and psychological aspects and influence of aerobic and resistance exercises among pSS patients. It was approved by the local research ethics committee on October 10, 2012, and it was conducted in the university’s exercise psychobiology laboratory (Brazil Platform; CEP: 125.852).

The eligible participants were ambulatory men or women with pSS in accordance with the European-American consensus group criteria of 2002.^19^ All participants signed an informed consent statement and were evaluated by a blinded physician. Serum and urine samples were collected and chest x-rays and echocardiograms were performed. Participants with pulmonary disease or heart failure were excluded. Patients taking rituximab or hypnotics were also excluded.

A visual analogue scale for pain (VAS-pain), the European League Against Rheumatism (EULAR) Sjögren’s Syndrome Patient Reported Index (ESSPRI) and the EULAR Sjögren’s Syndrome Disease Activity Index (ESSDAI) were used for assessing disease activity.

VAS-pain scales assess the severity of pain on a physical scale marked out from 0 to 100 millimeters, such that 0 represents “no pain” and 100 represents “the worst possible pain”. ESSPRI is a questionnaire in which the aim is to investigate the main symptoms of pSS patients in three domains: fatigue, pain and dryness. The scores are given by the patient by means of visual analogue scales (range from 0 to 10). The total score is the sum of the mean scores in these three domains.^20^ ESSDAI is a questionnaire completed by the physician that investigates pSS disease activity. It contains 12 domains relating to clinical and laboratory data (blood, immunological and urinary tests).^21^

To evaluate fatigue, we used the Profile of Fatigue and Discomfort - Sicca Symptoms Inventory (short form)(PROFAD-SSI-SF) and a visual analogue scale for fatigue (VAS-fatigue).

PROFAD-SSI-SF is used to characterize the fatigue pattern associated with Sjögren’s syndrome. It consists of nineteen questions that are separated into eight domains. PROFAD has nine questions split into four domains: cutaneous fatigue, mental fatigue, arthralgia and vascular, and SSI has ten questions split into four domains: ocular dryness, oral dryness, vaginal dryness, and cutaneous dryness. The scores can range from zero to seven, such that zero represents “the best” and seven represents “the worst”.^22^^,^^23^ The total score is the mean from summation of PROFAD and SSI and can range from 0 to 28. VAS-fatigue scales assess the severity of fatigue on a physical scale marked out from 0 to 100 millimeters, such that 0 represents “no fatigue” and 100 represents “the worst possible fatigue”.^24^

Sleep quality was evaluated using the Pittsburg sleep quality index (PSQI), in its version that has been validated for use in Portuguese, and using actigraphy for 15 days. We also used the Medical Outcomes Survey Short Form 36 (SF-36) for assessing quality of life.

PSQI is a questionnaire that consists of 19 self-rated questions and five questions that should be answered by bedmates or roommates. Each question contains seven components that are scored from 0 to 3 for assessing sleep quality and disturbances during the previous month. The sum of the seven components can range from 0 to 21. Scores ≥ 5 represent poor sleep quality and scores ≤ 4 represent good sleep quality.^25^ The PSQI components are as follows: subjective sleep quality, sleep latency, sleep duration, habitual sleep efficiency, sleep disturbances, use of sleep medication and daytime dysfunction.

Actigraphy is a technique used for assessing sleep-awake cycles. It enables recording of motor activities through limb movements.^16^^,^^17^ The sleep characteristics analyzed through actigraphy are the following: sleep latency, nightly awakenings (WASO) and sleep duration, which are all recorded in minutes; and sleep efficiency (regarding sleep latency), which is recorded as a percentage;

SF-36 is a questionnaire composed of 36 items that assesses functional capacity, pain, general health, vitality, social aspects, emotional aspects and mental health.^26^ Its scores range from 0 to 100.

### Statistical analysis

The data were summarized through descriptive statistics. For numerical variables, we used means and standard deviations, and minimum, median and maximum values. For categorical data, we used absolute and relative frequencies. The Spearman correlation test was used for analysis of correlations between variables and for comparisons among patients with sleep disorders and Sjögren’s syndrome. The correlations were classified as the following ranges: 0.00-0.19 “very weak”; 0.20-0.39 “weak”; 0.40-0.59 “moderate”; 0.60-0.79 “strong”; and 0.80-1.0 “very strong”.^27^ Student’s t test was used to analyze age and the Mann-Whitney test was used for the other variables (VAS-fatigue scale and PSQI). P < 0.05 was considered to be statistically significant. The analyses were carried out using the Minitab statistical software, version 13.1.

## RESULTS

### Descriptive analysis

Sixty-one patients who had previously been diagnosed as presenting pSS were initially assessed from 2015 to 2016. Eleven were excluded because they did not fulfill the inclusion criterion (a diagnosis of pSS in accordance with the 2002 criteria of the European-American consensus group),^19^ and one because of a diagnosis of heart disease. A total of 50 female patients with pSS were included in the study, with average age of 56.4 years, age range from 27 to 82 years and duration of syndrome symptoms ranging from 2 to 39 years (mean of 12 years of symptoms).

Regarding ESSDAI, the mean score was 2.3 and ranged from 0 to 12. After categorizing the score, 40 patients (80%) were found to present low disease activity (score < 5), and 10 (20%), moderate activity (scores between 5 and 14). None of them were classified as having high disease activity (score > 14). The ESSPRI and VAS-pain means were 6.31 (standard deviation, SD: 2.31) and 58 mm (SD: 2.8)

The total mean score for PROFAD-SSI-SF was 16.97 (SD: 6.23) and the mean for VAS-fatigue was 66 mm (SD: 2.7). In the fatigue domains, 75% of the participants presented high physical fatigue (PROFAD-physical > 2) and 65% reported having significant mental fatigue (PROFAD-mental > 2).

With regard to SF-36, in which the scores can range from 0 to 100 in each domain, the highest mean score was seen in the domain of functional capacity (mean = 61.5) and the lowest, in the domain of physical aspects (mean = 34.5). In the other domains, the means were as follows: 44.7 for pain, 56.3 for general health, 48.5 for vitality, 59.4 for social aspects, 48.6 for emotional aspects and 60.6 for mental health.

Regarding sleep quality measurement, the PSQI showed a total score of 8.9 (Table 1). When categorized, 13 patients (26%) had good sleep quality (score ≤ 5) and 37 patients (74%) had poor sleep quality (score > 5). Actigraphy indicated means of 26.2 minutes for sleep latency, 48.2 minutes for nightly awakening, 89.7% for sleep efficiency and 398.5 minutes (approximately 6.5 hours) for sleep duration (Table 2).

**Table 1.** Pittsburg sleep quality index measurement

Domain	Mean	Standard deviation
Sleep duration	1.1	1.2
Sleep disturbances	1.9	0.7
Sleep latency	1.5	1.2
Daytime sleepiness	1.5	0.9
Sleep efficiency	1.0	1.3
General quality of sleep	1.6	0.7
Use of drugs	0.8	1.3
Total score (sum)	8.9	4.7

**Table 2.** Actigraphy measurement

Domain	Mean	Standard deviation
Latency (minutes)	26.2	16.8
WASO (minutes)	48.2	39.7
Sleep efficiency (%)	89.7	8.4
Sleep duration (minutes)	398.5	80.9

WASO = wakings after sleep onset.

Table 3 presents a summary of correlations between PSQI and other variables. There were correlations, albeit weak, with the following: VAS-pain, VAS- fatigue, PROFAD-SSI and ESSPRI. In these cases, a positive correlation indicated that the higher the scale result for these variables was, the higher the total score for the PSQI also was. There were no correlations between PSQI and ESSDAI, between PSQI and duration of the symptoms or between PSQI and SF-36.

**Table 3.** Correlation between total score from Pittsburg sleep quality index (PSQI) and other variables

Variables	rs*	P-value
Duration of symptoms	0.084	0.562
EULAR Sjögren’s Syndrome Disease Activity Index	-0.091	0.531
Visual analogue scale for pain	0.329	0.020
Visual analogue scale for fatigue	0.381	0.006
Profile of Fatigue and Discomfort - Sicca Symptoms Inventory	0.308	0.030
EULAR Sjögren’s Syndrome Patient Reported Index	0.383	0.006
Short form-36	-0.166	0.248

*Spearman correlation.

Table 4 presents a summary of correlations between nightly awakenings (duration of WASO) and other variables. There was no correlation between the duration of WASO and PROFAD-SSI or between the duration of WASO and ESSPRI, but there was a weak correlation between the duration of WASO and ESSDAI, indicating that the higher the ESSDAI score was, the longer the duration of WASO also was.

**Table 4.** Correlation between duration of WASO (wakings after sleep onset) and other variables, shown through actigraphy

Variables	rs*	P-value
Profile of Fatigue and Discomfort - Sicca Symptoms Inventory	0.059	0.682
EULAR Sjögren’s Syndrome Patient Reported Index	-0.005	0.974
EULAR Sjögren’s Syndrome Disease Activity Index	0.352	0.012

*Spearman correlation.

## DISCUSSION

The present study confirmed that there was high prevalence of fatigue among pSS patients, in line with previous studies: one in which it was demonstrated that 96% of pSS patients suffered from significant physical fatigue (PROFAD-physical = 3.5) and another in which 48% of the patients reported having significant mental fatigue (PROFAD-mental = 2.8).^28^^,^^29^ Data obtained using multi-dimensional assessment tools showed that physical/somatic fatigue was more severe and more frequent among pSS patients and that, after controlling for depression, pSS patients were more fatigued than healthy controls, regarding general fatigue and physical fatigue, and they presented reduced activity in the MFI (Multifunctional Fatigue Inventory).^29^

It has also been reported that quality of life was worse among pSS patients and that direct healthcare costs in the pSS group were more than double those in the control group.^30^^,^^31^^,^^32^ A study conducted by Westhoff et al.^33^ confirmed this finding and demonstrated that pSS patients presented high levels of healthcare system usage, work disability and early retirement due to psychological and social factors (fatigue was included in those factors), but not glandular manifestations.

If fatigue rather than oral or ocular dryness causes increased healthcare usage and productivity losses, additional studies need to be carried out with the aim of bringing new insights into the mechanisms underlying fatigue, and the strategies that are required for addressing these common problems among pSS patients.

Our study showed significant positive correlations between sleep disorders and disease activity, as demonstrated through the correlation between actigraphy results and ESSDAI. Positive correlations were also found between the PSQI and the following variables: VAS-pain, VAS-fatigue, PROFAD-SSI score and ESSPRI.

A review by Abad et al.^12^ found that 75% percent of pSS patients complained of moderate or severe sleep disorders. Moreover, in comparison with rheumatoid arthritis patients, they had significantly higher sleep deficits (the difference between the need for sleep and actual duration of sleep), difficulty in falling sleep, increased muscle tension when trying to fall asleep, increased restless legs sensations, more nocturnal pain and more racing thoughts.^12^ The pSS group also complained of significantly more daytime sleepiness and fatigue and of not feeling rested after sleep,^1^ as confirmed by Gudbjornsson et al.^34^ using polysomnography. In the latter study, most of the patients made an association between daytime fatigue and sleep disorders.^34^

We found similar results, thus confirming the influence of sleep disorders on fatigue. We observed that only 13 patients (26%) indicated that they had good sleep quality in the PSQI, while 37 patients (74%) considered that their sleep was poor, predominantly with complaints regarding sleep quality.

According to Matuzakia et al., who studied sleep patterns in a sample of healthy adults living at the city of São Paulo, the sleep characteristic patterns observed through actigraphy were the following: sleep latency: 12.5 minutes (SD: 11); sleep efﬁciency: 80.6% (SD: 6.7); total duration of sleep: 365.4 minutes (SD: 57.4); and duration of wakings after sleep onset (WASO): 53.9 minutes (SD: 21.2).^18^ In our study, actigraphy showed that the means for sleep latency was 26.2 minutes. This is twice the time for healthy adults that was described by Matuzakia.^18^

Although sleep disorders among pSS patients had previously been demonstrated in other studies, with results similar to those found in our study, there is still a need for further study to clarify the relationship between fatigue and disease activity.^1^

### Study limitations

The limitation of the present study was that it had a descriptive design. A more appropriate study design would enable better investigation of the association between sleep disorders and fatigue and disease activity. Moreover, a cohort study on patients with high disease activity according to ESSDAI could be conducted in order to confirm our findings.

## CONCLUSION

The present study provides important information regarding a possible correlation between sleep disorders and disease activity. The study aimed to describe the characteristics of a group of patients with Sjögren’s syndrome, concerning fatigue, pain and sleep disorders. It could be seen that the subjects with the disease presented severe fatigue and sleep disorders. The results also demonstrated that sleep may have an influence on fatigue, and that there is an association between disease activity and sleep. These findings are of great clinical relevance, in view of the limited amount of information on this subject.

Therefore, we conclude that there is a need for proper control over disease activity and for development of strategies to help patients to sleep better in order to diminish their fatigue and improve their quality of life.

## References

[1] Mavragani CP, Moutsopoulos HM (2014). Sjögren syndrome. CMAJ.

[2] Fox RI (2005). Sjogren’s syndrome. Lancet.

[3] Peters JE, Isenberg DA (2012). Sjogren’s syndrome and association with other autoimmune and rheumatic diseases In: MR Casals, JH Stone, HM Moutsopoulos, editors. Sjögren’s Syndrome: Diagnosis and Therapeutics. Springer.

[4] Rehman H (2003). Sjögren’s syndrome. Yonsei Med J.

[5] Haldorsen K, Bjelland I, Bolstad AI, Jonsson R, Brun JG (2011). A five-year prospective study of fatigue in primary Sjögren’s syndrome. Arthritis Res Ther.

[6] Theander L, Strömbeck B, Mandi T, Theander E (2010). Sleepiness or fatigue? Can we detect treatable causes of tiredness in primary Sjögren’s syndrome?. Rheumatology (Oxford).

[7] Giles I, Isenberg D (2000). Fatigue in primary Sjögren’s syndrome: is there a link with the fibromyalgia syndrome?. Ann Rheum Dis.

[8] d’Elia HF, Rehnberg E, Kvist G (2008). Fatigue and blood pressure in primary Sjogren’s syndrome. Scand J Rheumatol.

[9] Strömbeck BE, Theander E, Jacobsson LT (2007). Effects of exercise on aerobic capacity and fatigue in women with primary Sjögren’s syndrome. Rheumatology (Oxford).

[10] Barendregt PJ, Visser MR, Smets EM (1998). Fatigue in primary Sjögren’s syndrome. Ann Rheum Dis.

[11] Mandl T, Hammar O, Theander E, Wollmer P, Ohlsson B (2010). Autonomic nervous dysfunction development in patients with primary Sjögren’s syndrome: a follow-up study. Rheumatology (Oxford).

[12] Abad VC, Sarinas PS, Guilleminault C (2008). Sleep and rheumatologic disorders. Sleep Med Rev.

[13] Ng WF, Bowman SJ (2010). Primary Sjögren’s syndrome: too dry and too tired. Rheumatology (Oxford).

[14] Hartkamp A, Geenen R, Bijl M (2004). Serum cytokine levels related to multiple dimensions of fatigue in patients with primary Sjögren’s syndrome. Ann Rheum Dis.

[15] Schutte-Rodin S, Broch L, Buysse D, Dorsey C, Sateia M (2008). Clinical guideline for the evaluation and management of chronic insomnia in adults. J Clin Sleep Med.

[16] Ancoli-Israel S, Cole R, Alessi C (2003). The role of actigraphy in the study of sleep and circadian rhythms. Sleep.

[17] Morgenthaler T, Alessi C, Friedman L (2007). Practice parameters for the use of actigraphy in the assessment of sleep and sleep disorders: an update for 2007. Sleep.

[18] Matuzaki L, Santos-Silva R, Marqueze EC (2014). Temporal sleep patterns in adults using actigraph. Sleep Sci.

[19] Vitali C, Bombardieri S, Jonsson R (2002). Classification criteria for Sjögren’s syndrome: a revised version of the European criteria proposed by the American-European Consensus Group. Ann Rheum Dis.

[20] Seror R, Ravaud P, Mariette X, Bootsma H, Theander E, Hansen A (2011). EULAR Sjögren’s Syndrome Patient Reported Index (ESSPRI): development of a consensus patient index for primary Sjögren’s syndrome. Ann Rheum Dis.

[21] Seror R, Theander E, Brun JG (2015). Validation of EULAR primary Sjogren’s syndrome disease activity (ESSDAI) and patient indexes (ESSPRI). Ann Rheum Dis.

[22] Bowman SJ, Booth DA, Platts RG, UK Sjögren’s interest group (2004). Measurement of fatigue and discomfort in primary Sjögren’s syndrome using a new questionnaire tool. Rheumatology (Oxford).

[23] Price DD, McGrath PA, Rafii A, Buckingham B (1983). The validation of visual analogue scales as ratio scale measures for chronic and experimental pain. Pain.

[24] Strömbeck B, Theander E, Jacobsson LT (2005). Assessment of fatigue in primary Sjögren’s syndrome: the Swedish version of the Profile of Fatigue. Scand J Rheumatol.

[25] Bertolazi AN, Fagondes SC, Hoff LS (2011). Validation of the Brazilian Portuguese version of the Pittsburgh Sleep Quality Index. Sleep Med.

[26] Ware JE, Sherbourne CD (1992). The MOS 36-item short-form health survey (SF-36). I. Conceptual framework and item selection. Med Care.

[27] Akoglu H (2018). User’s guide to correlation coefficients. Turk J Emerg Med.

[28] Segal B, Thomas W, Rogers T (2008). Prevalence, severity and predictors of fatigue in primary Sjogren’s syndrome. Arthritis Rheum.

[29] Godaert GL, Hartkamp A, Geenen R (2002). Fatigue in daily life in patients with primary Sjogren’s syndrome and systemic lupus erythematous. Ann N Y Acad Sci.

[30] Meijer JM, Meiners PM, Huddleston Slater JJ (2009). Health-related quality of life, employment and disability in patients with Sjogren’s syndrome. Rheumatology (Oxford).

[31] Segal B, Bowman SJ, Fox PC (2009). Primary Sjögren’s Syndrome: health experiences and predictors of health quality among patients in the United States. Health Qual Life Outcomes.

[32] Callaghan R, Prabu A, Allan RB (2007). Direct healthcare costs and predictors of costs in patients with primary Sjogren’s syndrome. Rheumatology (Oxford).

[33] Westhoff G, Dorner T, Zink A (2012). Fatigue and depression predict physician visits and work disability in women with primary Sjogren’s syndrome: results from a cohort study. Rheumatology (Oxford).

[34] Gudbjornsson B, Broman JE, Hetta J, Hällgren R (1993). Sleep disturbances in patients with primary Sjogren’s Syndrome. Br J Rheumatol.

